# Mechanisms and clinical outcomes of sacubitril-valsartan and SGLT2 inhibitors in heart failure patients: a retrospective cohort study

**DOI:** 10.3389/fcvm.2026.1931636

**Published:** 2026-10-20

**Authors:** Xuzheng Zhong, Lei Xia, Li Li, Bei Tang

**Affiliations:** 1 Department of Intensive Care Unit, The First People’s Hospital of Jiande, Jiande, China; 2 Department of Emergency, The First People’s Hospital of Jiande, Jiande, China

**Keywords:** heart failure, heart function, inflammatory level, sacubitril-valsartan, SGLT2 inhibitors

## Abstract

**Background:**

Heart failure represents a challenging cardiovascular disorder among the elderly, repeatedly exacerbating cardiac dysfunction and a reduced capacity for activity, thereby presenting significant therapeutic challenge.

**Objective:**

To analyse mechanisms of action and outcomes of combined therapy with sacubitril-valsartan and SGLT2 inhibitors for heart failure patients.

**Method:**

From October 2023 to October 2025, 150 heart failure patients were recruited and categorized them to the SV group (sacubitril-valsartan) and the SS group (sacubitril-valsartan and SGLT2 inhibitor) according to their treatment regimens. Both groups of patients received treatment with sacubitril-valsartan; the SS group additionally received SGLT2 inhibitor supplementation. The main parameters pre- and post- treatment were assessed and analyzed among both groups, including changes in cardiac function related indicators [left ventricle ejection fraction (LVEF), left ventricle end-diastolic diameter (LVEDD), left ventricle end-systolic diameter (LVESD), and cardiac output (CO)], serum indicators [β-endorphin (β-EP), cardiotrophin-1 (CT-1), N-terminal pro-B-type natriuretic peptide (NT-proBNP) changes], inflammatory indicators (IL-1β), interleukin-6 (IL-6), tumor necrosis factors-α (TNF-α), and clinical efficacy. Secondary indicators include heart failure quality of life scale scores, complication incidences, and adverse events incidences.

**Result:**

No marked discrepancy existed in the baseline clinical data among both groups (*P* > 0.05). Post-treatment, the indicators in both groups demonstrated remarkable improvement (*P* < 0.05). The LVEDD, LVESD, serum markers, inflammatory markers and MLHFQ scores in SS group were markedly below those in the SV group, whilst LVEF, CO and the overall clinical response rate were markedly increased (*P* < 0.05). The complications and adverse reactions incidences were markedly reduced in SS group vs. SV group (*P* < 0.05).

**Conclusion:**

The combined use sacubitril-valsartan and SGLT2 inhibitors demonstrates efficacy in improving cardiac function indicators, downregulate neuroendocrine and inflammatory factor levels, enhance clinical efficacy in heart failure patients, and can serve as an important reference for comprehensive treatment of such patients.

## 1 Introduction

Heart failure is a serious clinical syndrome that represents a terminal phase of numerous cardiac and vascular diseases. Its global prevalence remains high and is rising year on year due to an ageing workforce and increasing risk factor such as hypertension, posing a serious threat to human health and life (1). The etiology of heart failure is multifaceted and diverse, primarily resulting from the long-term progression of underlying cardiovascular diseases such as coronary artery diseases, cardiomyopathy, and myocarditis. Furthermore, chronic alcohol consumption, drug-induced damage and genetic factors may also precipitate heart failure (2). The core pathophysiological mechanisms involve myocardial remodelling, excessive activation of the neuroendocrine system, enhanced oxidative stress, disturbances in energy metabolism, and an imbalance in inflammatory responses. Among these, myocardial remodeling is a central pathological mechanism in heart failure progress, manifesting as cardiac hypertrophy, myocardial fibrillation, and heart chamber enlargement, ultimately leading to severely impaired systolic and diastolic cardiac functions (3). Typical clinical symptoms include exertional dyspnoea, dyspnoea at rest, fatigue, reduced exercise tolerance, lower limb oedema, chest tightness and shortness of breath, and paroxysmal nocturnal dyspnoea; in severe cases, acute pulmonary oedema, cardiogenic shock, and malignant arrhythmias may occur. This condition not only remarkably reduces patients' quality of life but also leads to extremely high readmission rates, placing a heavy healthcare workload on individuals and families. This represents a prominent public healthcare challenge in the cardiovascular field that requires urgent resolution, and is also one of the key areas of focus in current clinical and basic research (4).

**Table 1:** Baseline characteristics $\left(\bar{x}\pm s\right)$.

Parameters		SV group (*n* = 75)	SS group (*n* = 75)	95% CI		*t/x* ^2^	*P*
				Lower	Upper		
Age (year)		63.24 ± 7.03	63.72 ± 6.39	−2.647	1.687	−0.438	0.662
Weight (kg)		64.93 ± 3.86	64.43 ± 3.66	−0.711	1.716	0.819	0.414
Height (cm)		158.80 ± 4.47	158.22 ± 4.56	−0.873	2.039	0.791	0.430
Gender (male/female)		40/35	38/37	—	—	0.107	0.744
Body mass index (kg/m^2^)		25.82 ± 2.27	25.78 ± 1.83	−0.624	0.705	0.120	0.904
β-blocker use (*n*, %)		52/75	50/75			0.123	0.726
MRA use (*n*, %)		44/75	41/75			0.244	0.621
NYHA cardiac function grading [example, *n* (%)]	Grade I	28/75	30/75	—	—	0.112	0.737
	Grade II	35/75	36/75	—	—	0.027	0.870
	Grade III	12/75	9/75	—	—	0.498	0.480
Basic causes of heart failure [case, *n* (%)]	Coronary heart disease	28/75	30/75	—	—	0.112	0.737
	Hypertensive heart disease	34/75	36/75	—	—	0.107	0.743
	Dilated cardiomyopathy	13/75	9/75	—	—	0.852	0.356
Combined underlying diseases [example, *n* (%)]	Type 2 diabetes	29/75	30/75	—	—	0.082	0.868
	Hypertension	34/75	36/75	—	—	0.107	0.743
	Coronary artery disease	12/75	9/75	—	—	0.498	0.480
Smoking history (Yes/No)		56/19	55/20	—	—	0.035	0.852
Drinking history (Yes/No)		53/22	51/24	—	—	0.125	0.723

**Table 2:** Changes in indicators related to cardiac function $\left(\bar{x}\pm s\right)$.

Parameter	Time	SV group	SS group	95% CI		*t*	*P*
				Lower	Upper		
LVEF (%)	Pre-treatment	37.42 ± 2.95	37.06 ± 2.45	−0.519	1.231	0.804	0.423
	Post- treatment	48.47 ± 3.78*	53.08 ± 4.84*	−6.019	−3.213	−6.504	<0.001
LVEDD (mm)	Pre-treatment	56.54 ± 3.82	56.28 ± 3.87	−0.978	1.504	0.419	0.676
	Post- treatment	50.11 ± 3.14*	46.68 ± 2.89*	2.455	4.400	6.961	<0.001
LVESD (mm)	Pre-treatment	43.83 ± 2.71	43.63 ± 2.88	−0.716	1.091	0.410	0.682
	Post- treatment	37.98 ± 2.01*	35.62 ± 2.07*	1.703	3.019	7.091	<0.001
CO (L/min)	Pre-treatment	3.19 ± 0.73	3.32 ± 0.77	−0.377	0.110	−1.083	0.281
	Post- treatment	4.09 ± 0.74*	4.96 ± 0.85*	−1.131	−0.617	−6.712	<0.001

* Indicates a statistically significant difference vs. pre-treatment within the same group (paired-samples *t*-test, *P* < 0.05).

**Table 3:** Serum indicators $\left(\bar{x}\pm s\right)$.

Parameter	Time	SV group	SS group	95% CI		*t*	*P*
				Lower	Upper		
β-EP (pg/mL)	Pre-treatment	143.19 ± 14.77	140.99 ± 14.31	−2.493	6.891	0.926	0.356
	Post- treatment	108.21 ± 10.31*	72.67 ± 7.22*	32.666	38.410	24.455	<0.001
CT-1 (pg/mL)	Pre-treatment	321.17 ± 28.12	323.73 ± 32.51	-12.366	7.250	-0.515	0.607
	Post- treatment	221.35 ± 18.39*	152.97 ± 13.85*	63.126	73.633	25.720	<0.001
NT-proBNP (pg/mL)	Pre-treatment	1,791.43 ± 269.40	1,775.19 ± 260.59	-69.290	101.760	0.375	0.708
	Post- treatment	882.26 ± 152.61*	380.14 ± 112.42*	458.863	545.363	22.942	<0.001

* Indicates a statistically significant difference vs. pre-treatment within the same group (paired-samples *t*-test, *P* < 0.05).

**Table 4:** Inflammatory indicators.

Parameter	Time	SV group	SS group	95% CI		*t*	*P*
				Lower	Upper		
IL-1β (ng/L)	Pre-treatment	65.14 ± 8.25	63.72 ± 8.41	-1.265	4.111	1.046	0.297
	Post- treatment	42.89 ± 3.85*	34.22 ± 3.56*	7.467	9.860	14.305	<0.001
IL-6 (ng/L)	Pre-treatment	35.25 ± 3.61	34.76 ± 4.50	-0.826	1.806	0.736	0.463
	Post- treatment	22.37 ± 2.62*	12.77 ± 2.89*	8.713	10.493	21.313	<0.001
TNF-α (ng/L)	Pre-treatment	29.52 ± 3.97	29.87 ± 3.72	-1.596	0.888	-0.563	0.575
	Post- treatment	15.72 ± 3.13*	10.72 ± 2.68*	4.065	5.945	10.525	<0.001

* Indicates a statistically significant difference vs. pre-treatment within the same group (paired-samples *t*-test, *P* < 0.05).

**Table 5:** Clinical efficacy [*n*(%)].

Group	Obvious effect (*n*)	Effective (*n*)	Ineffective (*n*)	Total effective rate (*n*, %)
SV group	30	33	12	63 (84.00)
SS group	34	36	5	70 (93.33)
*x* ^2^	3.979			
*P*	<0.05			

**Table 6:** MLHFQ scores $\left(\bar{x}\pm s,\mathrm{scores}\right)$.

Time		SV group	SS group	95% CI		*t*	*P*
				Lower	Upper		
Pre-treatment		50.66 ± 5.33	49.42 ± 5.54	−0.509	2.999	1.403	0.163
Post- treatment		25.37 ± 2.89	20.14 ± 2.27	4.396	6.072	12.342	<0.001
95% CI	Lower	23.911	27.918				
	Upper	26.678	30.649				
*t*		36.126	42.376				
*P*		<0.001	<0.001				

Currently, the treatment of heart failure has evolved into an integrated model comprising “etiological treatment, symptomatic treatment and comprehensive management”, with the core therapeutic objectives being to improve cardiac function, reverse myocardial remodelling, decrease the risks of unfavorable cardiac events, increase quality of life, and prolong life expectancy (5). Treatment approaches primarily include lifestyle interventions (weight control, low-salt diet, moderate exercise, smoking cessation and alcohol restriction), aetiological treatment (managing fundamental conditions such as elevated blood pressure), and pharmacological and non-pharmacological therapies (heart resynchronization treatment, implanted cardioverter-defibrillator, heart transplant, etc.) (6, 7). Among these, pharmacotherapy is a fundamental and crucial component, running throughout the entire treatment process for heart failure. Traditional drug therapies primarily include ace inhibitor drugs, antagonists of the vasopressin II receptors, β-blocker, and mineralocorticoid antagonists. Although these can alleviate patients' clinical symptoms and delay disease progression to some extent, a proportion still experience poor treatment outcomes and recurrent deterioration, and these therapies fail to effectively reduce readmission rates and mortality (8). In recent years, the development and clinical application of novel anti-heart failure drugs have brought about breakthroughs in heart failure therapy. Among these, sacubitril-valsartan and sodium-glucose cotransporter-2 (SGLT2) inhibitor, owing to their unique mechanisms of action and significant therapeutic effects, have gradually become first-line drugs on clinical heart failure therapy and have emerged as the research focus in the cardiovascular field the recent years (9).

Sacubitril-valsartan is a newly developed angiotensin receptor-neuropeptidase inhibitors. Unlike traditional anti-heart failure drugs, it exerts comprehensive cardiovascular protective effects through its double-acting mechanisms, representing a major breakthrough in the management of chronic heart disease (10). On the one hand, it specifically blocks angiotensin II receptors, thereby suppressing excessive activity of renin-angiotensin-aldosterone axis. This reduces vasoconstriction and water-sodium retaining, while also suppressing cardiac fibrosis and remodelling, thus alleviating myocardial damage and improving cardiac function (11). On the other hand, it inhibits the activity of enkephalinase, elevating levels of vasopressin-sensitive peptides in the body, thereby exerting vasodilatory, natriuretic and diuretic effects and inhibiting sympathetic nerve activation. This further reduces preload and afterload on the heart, improves myocardial blood and oxygen supply, and alleviates symptoms such as dyspnoea and oedema in heart failure patients (12). SGLT2 inhibitors were initially used for glycaemic control in type 2 diabetes, but numerous clinical studies have confirmed their substantial cardioprotective effects in recent years, and they have been incorporated into guidelines for the treatment of heart failure (13). Their mechanism of action has several distinctive features. Firstly, through their inhibitory effect on glucose reabsorption in proximal tubules, they produce an osmotic diuretic effect, thereby reducing preload on the heart. Concurrently, they improve myocardial energy metabolism, promote the utilisation of ketone bodies for energy by cardiomyocytes, and reduce myocardial cell damage (14). Furthermore, these drugs can also inhibit oxidant stress and inflammation, reducing secretion of inflammatory growth factors and thereby alleviating myocardial inflammation and fibrosis (15). Both of them have important clinical value in treating heart failures, but specific mechanisms of action, synergistic effects, and clinical differences of their combined or individual use still need to be further explored and validated through large-scale retrospective studies.

Against the backdrop of the aforementioned research, the central hypotheses guiding this study are: the combined use of acubitril valsartan and an SGLT2 inhibitor through synergistic effects can markedly improve cardiac function in heart failure patients and reduce adverse cardiovascular events. The anticipated finding is that, following treatment, cardiac function parameters in the combination therapy group show markedly superior improvement relative to the monotherapy group. Additionally, the combination of the two drugs had good safety and no significant serious adverse reactions. The findings of this study have remarkable potential clinical applications: they offer direct evidence supporting the mechanism of synergistic action in the combination of sacubitril/valsartan and SGLT2 inhibitors; clarify the role of these two drugs—either in combination or when used alone—in heart failure therapy; and provide reliable, evidence-based medical support for optimization of heart failure treatment regimens, precise selection of medication strategies, and the implementation of personalized treatment in clinical practice. This method has valuable clinical applications and research significance in improving prognosis for heart failure patients and reducing medical burden, and can also provide reference and inspiration for subsequent related research.

## 2 Materials and methods

### 2.1 Study design

This retrospective observational cohort study investigates the mechanisms and clinical efficacy of sacubitril/valsartan and SGLT2 inhibitors in heart failure patients. The research subjects selected 150 heart failure patients during October 2023 to October 2025, and categorized them to SV group (sacubitril-valsartan treatment group) and SS group (sacubitril-valsartan and SGLT2 inhibitor combination treatment group) depending on the actual clinical therapy regimen each patient received. Both groups received treatment with sacubitril-valsartan, while the SS group also received an SGLT2 inhibitor in conjunction with the aforementioned treatment. This study gathered patient data meeting the inclusion standards via the hospital medical record system and other sources. A total of 165 potential patients were enrolled for eligibility screening. After applying the predefined exclusion standards, 158 patients were finally included in the study cohort. All enrolled patients received unified follow-up. Eight patients were lost to follow-up during the observation period. Ultimately, 150 patients completed data acquisition and statistical analysis. The primary objective of the present study is to evaluate and contrast the clinical effectiveness of the “sacubitril-valsartan + SGLT2 inhibitor” treatment regimen with the sacubitril-valsartan alone treatment regimen in patients with heart failure, and establish an evidence-based medicine for clinical management in such patients. See Figure 1 for details of the case selection and follow-up procedures.

**Figure 1:**
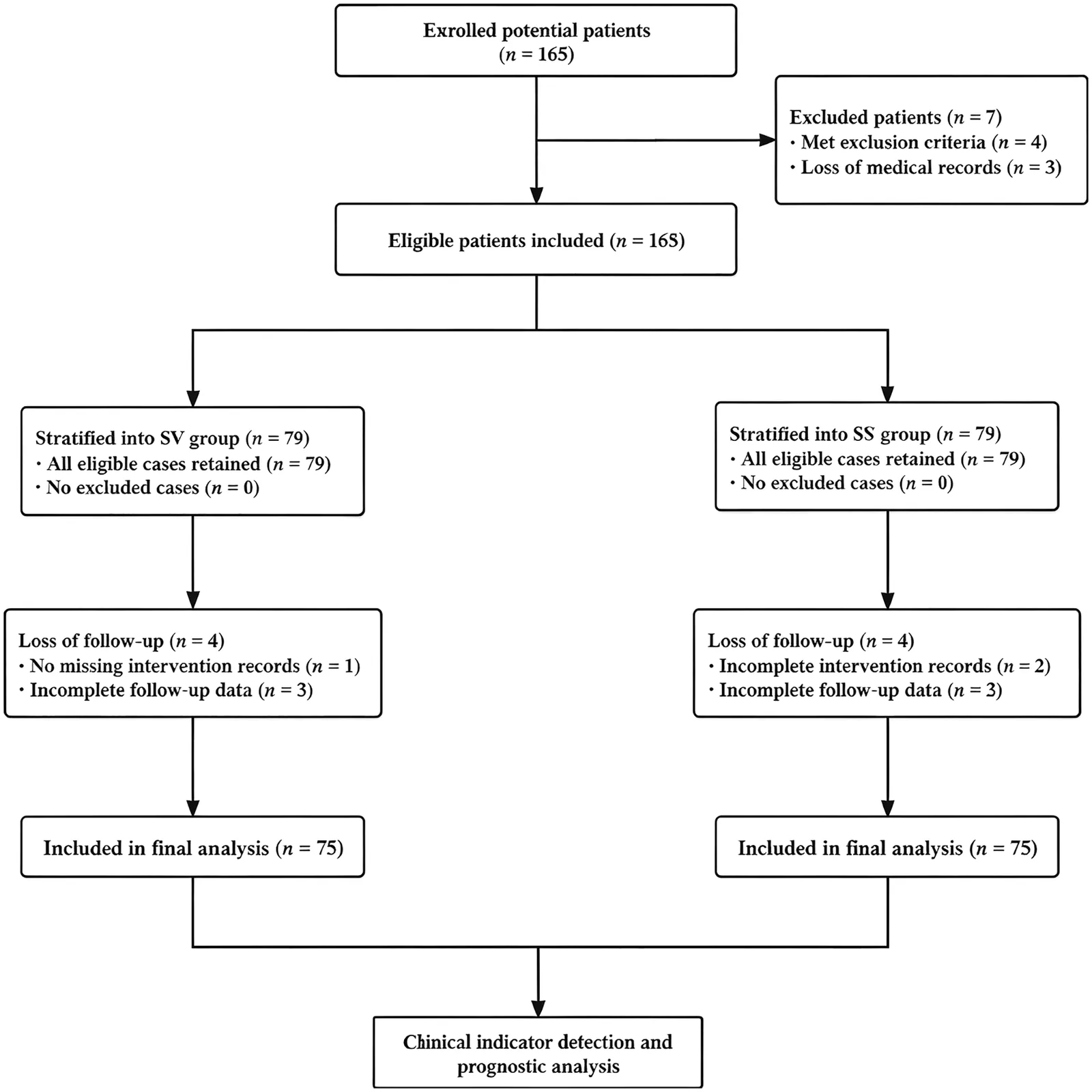
Flowchart.

### 2.2 Blinded outcome assessment

This is a retrospective observational cohort study with blinded outcome assessment. Since patients' treatment regimens were already determined in real-world clinical practice prior to this analysis, patients and treating clinicians could not be blinded to treatment assignment. Only outcome assessors were blinded to group allocation to minimize evaluation bias. Specifically, an independent data-management team encoded clinical data from the two groups. Medication-related information was removed from source medical records, whereas objective data including baseline characteristics, changes in cardiac function and complications were retained. Outcome assessors evaluated treatment efficacy and safety exclusively using the encoded datasets following pre-specified criteria. Group allocation information was unblinded only after all statistical analyses had been finalized. This approach helps reduce subjective judgement and improves the reliability of outcome evaluation.

### 2.3 Inclusion and exclusion criteria

Inclusion criteria: (1) meets the diagnostic criteria outlined in “Chinese Guideline on Diagnosis and Heart Failure Treatment” (16); (2) ages: 40–75; all genders welcome; (3) no use of sacubitril-valsartan or SGLT2 inhibitors during the 3 months prior to group entry; (4) patient compliance was good, and they cooperated in completing the 6-month follow-up and all relevant tests; (5) individuals able to tolerates the medications used; (6) individuals capable of clearly describing their symptoms and cooperating in completing the life questionnaire qualities.

Exclusion criteria: (1) patients suffering from severe heart, kidney, or other organs failure; (2) individuals who are allergic to the drug ingredients used in this study; (3) individuals suffering from pre-existing conditions that may affect immunocompetence (such as malignancies, hematological disorders, etc.); (4) individuals diagnosed with severe intellectual disabilities, mental health conditions, or who are unable to cooperate during follow-up visits; (5) individuals expected to have a high rate of loss to follow-up due to anticipated poor adherence (voluntary self-discontinuation of medications for personal reasons), moving out of the study area, or other personal reasons; (6) patients with objective confounding clinical conditions including recent major surgery, active severe infection, terminal malignancy or other acute critical illnesses that may independently interfere with study outcomes and follow-up safety; (7) other potentially influencing factors for follow-up outcome parameters.

### 2.4 Ethical statement

This study strictly adheres to the Helsinki Declarations and hospital's ethical guidelines, and has been approved through the hospital ethics committee. This retrospective medical-record-based study was not registered in any public clinical trial registry. The predefined inclusion-exclusion criteria, outcome measures and statistical plan were established and documented during the ethical review process prior to data collection.

### 2.5 Treatment measures

Patients in both groups received standardized baseline heart-failure-related therapy as part of their routine clinical management, including heart-related health education, medication compliance management, volume status control, and monthly home-self-care sessions routinely provided in clinical practice. Individualized heart-failure dietary plans formulated by nutritionists (strict sodium and daily fluid intake control), individualized moderate-intensity aerobic exercise advice, and standardized daily-life management (7–8 h sleep per day, smoking cessation, alcohol restriction and body-weight control) were clinical measures implemented during their real-world treatment, rather than interventions specially assigned for this retrospective study.

SV group (sacubitril-valsartan treatment group): In addition to the unified basic treatment plan mentioned above, sacubitril-valsartan sodium tablets were used for intervention. sacubitril-valsartan sodium tablets (50 mg × 28 tablets, national drug approval number J20171054) have an initial dose of 50 mg, taken orally twice a day after breakfast and dinner. Based on the patient's blood pressure, cardiac function grading, renal function, and blood potassium tolerance, dose titration is performed every 2 weeks, gradually increasing to 100 mg twice a day to maintain treatment. Individuals at risk of low systolic blood pressure, renal insufficiency, and hyperkalemia should temporarily postpone dose escalation and maintain tolerance to the maximum dose (200 mg, twice daily). Ensure that the patient's systolic blood pressure is ≥95 mmHg throughout the entire medication treatment process, and adjust the dosage to the maximum tolerated dose within 4 weeks after medication.

SS group (sacubitril-valsartan and SGLT2 inhibitor combination therapy group): SGLT2 inhibitor was added to the same treatment regimen as sacubitril-valsartan in the SV group. Dapagliflozin tablets (10 mg × 14 tablets) are to undergo oral once daily at a dose of 10 mg, 30 min following breakfast. Both groups received study-related drug therapy continuously for 3 months. Patients remained on guideline-directed heart-failure maintenance therapy thereafter. Serum indicators were assessed at 3-months after treatment initiation, whereas echocardiographic cardiac-function parameters were evaluated at the 6-month follow-up after treatment initiation. Monthly safety monitoring was performed throughout the whole observation period. The attending physician may adjust symptomatic supportive treatment based on the condition and tolerance.

### 2.6 Observation indicators

Major indicators.

#### 2.6.1 Changes in cardiac function-related indicators

Color Doppler ultrasound (EPIQ7C, Philips Ultrasound, USA) assessed changes in cardiac function-related parameters in patients before treatment and 6 months after treatment (17). Place the patient in the left lateral decubitus positioning, instruct them to breathe calmly and hold their breath during the examination, and scan utilizing a 2.5–3.5 MHz matrix array probe. Standard left ventricular parasternal long-axis images are displayed, and M-mode ultrasound is used to measure left ventricle ejection fraction (LVEF), left ventricle end-diastolic diameter (LVEDD), left ventricle end-systolic diameter (LVESD), and cardiovascular output (CO). All measured values are averaged over three full cardiac cycles to minimize error.

#### 2.6.2 Serum indicators

Collect approximately of fasting peripheral blood from the subjects at baseline before treatment and 3 months after treatment. Place the blood sample in a pre cooled anticoagulant blood collection tube containing EDTA (Ethylene Diamine Tetraacetic Acid, 1 g/L) and aprotinin (0.5 U/L), mix well, centrifuge for 15 min at 3,000 r/min to isolate the upper layer of plasma, and store it frozen at −20 °C for future use. This study detected the levels of serum β-endorphin (β-EP), cardiotrophin-1 (CT-1), and N-terminal pro-B-type natriuretic peptide (NT-proBNP) before with after treatment. Detection of β-EP and CT-1 utilizing the enzyme-linked immunosorbent assay (ELISA) (CSB-E06821h, CUSABIO, China and EK0563, Boster, China) (18, 19), while NT-proBNP was detected using a 91001 Ruipu fluorescence dry quantitative analyzer produced by Response Biomedical Corporation in Canada. All indicator tests are strictly carried out in accordance with the corresponding reagent kit and instrument standard operating procedures, and experimental quality control is carried out synchronously to ensure accurate and reliable test results.

#### 2.6.3 Inflammatory indicators

Inflammatory cytokine levels were measured in both groups of participants (20). A 5-mL blood sample was collected from the antecubital vein early in the morning on empty stomach and processed by centrifugation at 3,000 r/min for 10 min. After separating the serum, the supernatant was collected. Our hospital's laboratory uniformly uses the ELISA method to test serum samples and records patients' postsurgical serum inflammation marker levels. The human interleukin-1β (IL-1β) ELISA kit (PI305), human interleukin-6 (IL-6) ELISA kit (PI325) and human tumour necrosis factor-α (TNF-α) ELISA kit (97072ES96) were used to analyse the levels of IL-1β, IL-6 and TNF-α in serum. The kits were purchased from Shanghai Beyotime Biotechnology Co., Ltd. and Shanghai Yeasen Biotechnology Co., Ltd.

#### 2.6.4 Clinical efficacy

The clinical efficacy is divided into three levels: obvious effect, effective, and ineffective. Significant effect: LVEF increased by ≥5% comparing to before treatment, LVEDD and LVESD decreased by ≥10% compared to before treatment, CO remarkably improved, serum NT-proBNP, CT-1, β-EP, inflammatory indicators levels decreased by ≥30% compared to before treatment. Effective: LVEF increased by 2%–4% compared to before treatment, LVEDD and LVESD decreased by 5%–9% compared to before treatment, CO improved, and serum NT-proBNP, CT-1, β-EP, inflammatory indicators levels decreased by 15%–29% compared to before treatment. Ineffective: No improvement or even deterioration in echocardiographic parameters related to cardiac function; no significant reduction or an increase in serum NT-proBNP, CT-1, β-EP and inflammatory factors levels compared with pre-treatment levels; and no relief or even worsening of clinical symptoms related to heart failure.

Secondary indicators.

#### 2.6.5 Quality of life scores

The Minnesota Life with Heart Failure Questionnaires (MLHFQ) was used to quantitatively evaluate patients' quality of life, with assessments conducted at before and after treatment (21). This questionnaire comprises 21 items covering multiple dimensions, including physical activity, emotional state, social interaction and disease-related symptoms. Each item is evaluated on a 5-point numerical rating scale of 1–5 (where 1 indicates no related distress and 5 indicates extremely severe distress), with the overall score ranges from 0 to 105. The lower the score, indicating a higher quality of life for the patient.

#### 2.6.6 Complications

This study mainly observed and statistically analyzed the occurrence of complications during the treatment period, with monitoring indicators including sudden cardiac death, readmission due to sudden cardiac death, stroke, and severe arrhythmias (including torsades de pointes). During the treatment period, a systematic evaluation will be conducted via monthly clinic visits, phone follow-ups, and review of hospital records to dynamically observe whether patients have the clinical manifestations of the above-mentioned complications. Routine rechecks of electrocardiograms and echocardiograms are conducted at 2 months. If patient presents suspicious clinical symptom or auxiliary examinations indicate abnormal signs, further tests including coronary CT angiography and head CT scans shall be performed immediately to confirm the diagnosis. All complication cases were independently judged by two attending cardiologists in accordance with the relevant standards of the “Chinese Guideline to Diagnosis and Heart Failure Treatments”. The time, type, and severities of the complication was clearly defined. If the differences in the evaluation results between the two physicians, the final review and ruling would be made by the deputy chief physician or a physician with a higher professional title to department. The overall incidence of complications was summarized and compared throughout the duration of therapy in both groups.

#### 2.6.7 Adverse reactions

This study observed drug-related adverse reactions in patients during treatment, mainly including symptoms of insufficient blood volume including symptoms of hypovolemia and adverse events such as urinary tract and genital tract infections. During treatment, at monthly follow-up visits, investigators systematically asked patients about their symptoms using a standardized adverse event assessment tool. If there were suspected adverse reactions, relevant examinations such as blood potassium, urine routine, and urine culture were promptly completed to assist in differential diagnosis. Finally, adverse reaction rates were calculated for both groups of patients during the therapy period, and the overall incidence rate was determined.

### 2.7 Follow-up visits

Utilized clinical diagnosis and treatment records from inpatients and treatment archive data, systematically extracting patient follow-up data after treatment, collecting follow-up data at 6 months after treatment, and dynamically evaluating the improvement effects of sacubitril-valsartan monotherapy and combination with SGLT2 inhibitor regimen on cardiac functions, serum biological indicators, and inflammatory markers in heart failure patients based on cardiac ultrasound results. During the follow-up process, drug related adverse reactions, were synchronously observed and recorded, while the occurrence of cardiovascular complications such as sudden cardiac death was also recorded. This study is limited by the retrospective study itself, and the integrity of follow-up data depends on the original medical records. This study established rigorous, standardized criteria for case selection and data extraction, including only cases with complete clinical data at every observation time point to maximize the accuracy and reliable nature of the data. This provides an evidence-based foundation for analyzing the clinical effectiveness and long-term efficacy of the combination therapy of sacubitril-valsartan and an SGLT2 inhibitor in the management of heart failure.

### 2.8 Sample size calculation

We used G*Power 3.1 software to conduct a power analysis to calculate the sample size required to detect statistical significance. Considering the primary clinical outcomes and previous studies (22), and taking into account a significance level of 0.05% and 85% power, we calculated that 59 patients were needed per group; to account for potential uncertainties, the final sample size for this study was set at 75 patients per group. We determined that the sample sizes for this study were sufficient to draw reliable conclusions.

### 2.9 Statistical methods

Data analysis was performed using SPSS 28.0 statistical software. Flowcharts were created using Lucidchart. All datasets in this study were tested for normality. Baseline characteristics and continuous outcome variables, including cardiac function-related parameters, serum markers, inflammatory markers, and quality-of-life scores, are presented as mean ± standard deviation $\left(\bar{x}\pm s\right)$. For within-group comparisons of pre- vs. post-treatment changes, paired-samples *t*-tests were used to evaluate longitudinal changes within each group. For between-group comparisons, independent-samples *t*-tests were applied to compare outcome indicators between the SV and SS groups at the same time point. Clinical efficacy, complication rates, and adverse reaction rates are reported as percentages (%), and inter-group comparisons were performed using the *χ*^2^ test. All statistics were two-sided; a *P* value <0.05 was considered clinically significant.

## 3 Results

### 3.1 Basic information

This study is a retrospective observational cohort study that included 150 heart failure patients between October 2023 and October 2025. According to the treatment protocol, the subjects were divided into the SV group and the SS group to investigate the action mechanisms and clinical efficacy of sacubitril-valsartan and SGLT2 inhibitors in heart failure. Prior to the start of the study, we collected and recorded in detail the baseline demographics and clinically relevant data for both groups. The findings revealed no marked discrepancy existed in baseline characteristics between both groups (*P* > 0.05). These results ensure that clinical confounding factors were effectively controlled during the study, reducing underlying interference with the study conclusions and laying dependable groundwork for an accurate assessment of the clinical effectiveness of sacubitril-valsartan monotherapy and combination therapy with an SGLT2 inhibitor for heart failure.

Major outcome.

### 3.2 Heart function related indicators

The monitoring of cardiac function related ultrasound indicators in this study revealed that no marked discrepancy existed in LVEF, LVEDD, LVESD, and CO of SV and SS groups pre-treatment (all *P* > 0.05). This indicated that cardiovascular function and ventricular structure were well matched between both groups pre-treatment, and no marked bias in baseline data across groups, indicating good clinical comparability. After different intervention treatments, both groups revealed significant improvement in the above-mentioned cardiac function and ventricular structure related indicators compared to pre-treatment, and marked discrepancy observed between both groups post-treatment (both *P* < 0.05). The LVEF, LVEDD, LVESD, and CO of the SV group were (48.47 ± 3.78)%, (50.11 ± 3.14) mm, (37.98 ± 2.01) mm, and (4.09 ± 0.74) L/min, respectively, while those of the SS group were (53.08 ± 4.84)%, (46.68 ± 2.89) mm, (35.62 ± 2.07) mm, and (4.96 ± 0.85) L/min, respectively post-treatment. The results revealed that both treatment regimens were effective in reducing the diastolic and systolic end-diastolic diameters of patient's ventricle, improving state of ventricular remodeling, enhancing the patient's cardiac output and ejection function. Compared to SV group, SS group demonstrated more significant and advantageous effects on improving the patient's cardiac structural remodeling and cardiac function.

### 3.3 Serum indicators

The results of serum biochemical marker detection in this study revealed that no marked discrepancy existed in the levels of serum β-EP, CT-1, and NT-proBNP between the SV group and SS group pre-treatment (all *P* > 0.05). This indicated that baseline levels of neuroendocrine activation and heart failure related markers were similar between both groups, and the baseline data were balanced and comparable. After corresponding intervention plans, the levels of the three indicators mentioned above in two groups were remarkably reduced as measured against pre-treatment, and marked discrepancy observed between pre- and post-treatment in same group (*P* < 0.05). There were markedly distinct differences in the indicators across all groups post-treatment (all *P* < 0.01). The SV group had β-EP, CT-1, and NT-proBNP levels of (108.21 ± 10.31) pg/mL, (221.35 ± 18.39) pg/mL, and (882.26 ± 152.61) pg/mL, respectively. The SS group had levels of (72.67 ± 7.22) pg/mL, (152.97 ± 13.85) pg/mL, and (380.14 ± 112.42) pg/mL, respectively. The above results suggest that both treatment options can effectively inhibit neuroendocrine overactivation and downregulate the expression of heart failure markers, and the SS group has a better inhibitory and improvement effect on relevant indicators than the SV group.

### 3.4 Inflammatory indicators

The monitoring of serum inflammatory factor indicators in this study revealed that no marked discrepancy existed (*P* > 0.05) in the inflammatory factor indicators levels between both groups pre-treatment. The baseline inflammatory response levels of both groups are basically the same, and the baseline data is balanced and comparable, with a good basis for inter group comparison. After treatment with different regimens, the levels of the above-mentioned inflammatory factors in both groups remarkably decreased as measured against pre-treatment, and marked discrepancy observed between pre- and post-treatment in same group (both *P* < 0.05). Among them, IL-1β, IL-6, and TNF-α in the SV group were (42.89 ± 3.85) ng/L, (22.37 ± 2.62) ng/L, and (15.72 ± 3.13) ng/L, respectively. The SS groups were (34.22 ± 3.56) ng/L, (12.77 ± 2.89) ng/L, and (10.72 ± 2.68) ng/L, respectively. There were markedly distinct differences in the indicators across all groups post-treatment (all *P* < 0.01). The results indicate that both treatment regimens can effectively reduce the inflammatory factors levels in body, alleviate the inflammatory stress response, and SS group has a remarkably greater improvements effect on inflammation levels than the SV group.

### 3.5 Clinical efficacy

The outcomes of the clinical efficacy evaluation revealed that among the 75 patients in the SV group, 63 responded to treatment, representing a total clinical response rate of 84.00%; among the 75 patients in the SS group, 70 responded to treatment, representing a total clinical response rate of 93.33%. The marked discrepancy observed in total clinical effective rate of both groups (*P* < 0.05). This indicates that the SS treatment regimen was markedly superior to the SV regimen in terms of its comprehensive clinical efficacy in improving the clinical symptoms and overall conditions with heart failure.

Secondary outcome

### 3.6 Quality of life score

The MLHFQ quality of life scores monitoring in this study revealed that no marked discrepancy existed in MLHFQ scores of both groups pre-treatment (*P* > 0.05). The baseline quality of life levels of both groups are similar, and the baseline data were interchangeable. After different intervention plans, both groups revealed a markedly decrease in MLHFQ scores compared to pre-treatment, and marked discrepancy observed of pre- and post-treatment in same group (*P* < 0.05). The MLHFQ score for the SV group was (25.37 ± 2.89) scores, while for the SS group it was (20.14 ± 2.27) scores. The marked discrepancy observed in scores between groups post-treatment (*P* < 0.01). The results indicate that both treatment options can improving life quality cores, and SS group has a better improvement effect than the SV group.

### 3.7 Complications

The statistical outcomes of complications are displayed in Table 7. Among the 75 subjects in SV group, 11 experienced related complications, resulting in an overall incidence rate of 14.67%; in contrast, among the 75 subjects in SS group, only 4 experienced complications, representing in an overall incidence rate of 5.33%. The marked discrepancy observed difference in the overall complications rate of both groups (*P* < 0.05). When conducting clinical interventions for patients with heart failure, the complications rate in SV group was remarkably higher than SS group.

**Table 7:** Complications [*n*(%)].

Complications	SV group	SS group	*x* ^2^	*P*
Sudden cardiac death	2 (2.67)	1 (1.33)	1.020	0.312
Readmission due to heart failure	3 (4.00)	1 (1.33)	1.846	0.174
Acute myocardial infarction	2 (2.67)	1 (1.33)	1.020	0.312
Stroke	2 (2.67)	0 (0.00)	3.046	0.081
Severe arrhythmia	2 (2.67)	1 (1.33)	1.020	0.312
Total incidence rate	11 (14.67)	4 (5.33)	5.556	<0.05

**Table 8:** Adverse reactions [*n*(%)].

Adverse reactions	SV group	SS group	*x* ^2^	*P*
Dizziness	2 (2.67)	1 (1.33)	1.020	0.312
Orthostatic Hypotension	2 (2.67)	1 (1.33)	1.020	0.312
Frequent urination	2 (2.67)	1 (1.33)	1.020	0.312
Urgent need to urinate	1 (1.33)	1 (1.33)	0.000	1.000
Nausea	2 (2.67)	0 (0.00)	3.046	0.081
Diarrhea	2 (2.67)	1 (1.33)	1.020	0.312
Liver dysfunction	1 (1.33)	1 (1.33)	1.020	0.312
Muscle pain	2 (2.67)	1 (1.33)	1.020	0.312
Hyperkalemia	1 (1.33)	0 (0.00)	1.005	0.316
Total incidence rate	15 (20.00)	7 (9.33)	4.880	<0.05

### 3.8 Adverse reactions

The statistical results of adverse reactions in this study revealed that 15 out of 75 subjects in the SV group experienced related adverse reactions, with a total incidence rates of 20.00%. Among the 75 subjects in SS group, 7 experienced adverse reactions, representing in an overall incidence rate of 9.33%. The marked discrepancy observed in the total incidences of adverse reactions of both groups (*P* < 0.05). When implementing clinical drug interventions for heart failure patients, the drug tolerance and overall safety of the SS group treatment plan are remarkably better than those of the SV group.

## 4 Discussion

Heart failure is a key risk factors for mortality in cardiovascular disease patients, characterised by a complex pathophysiology and a progressive, irreversible course (23). The core pathophysiological changes in this condition are primarily characterised by ventricular remodelling, excessive neuroendocrine activation, and a persistent chronic inflammatory response (24). Following myocardial damage, the body's renin-angiotensin-aldosterone systems and sympathetic nervous systems are abnormally activated, resulting in the massive release to neuroendocrine mediators such as β-EP and NT-proBNP, which further exacerbate myocardial fibrosis and ventricular structural remodelling. Concurrently, inflammatory mediators persistently infiltrate myocardial tissue, inducing a chronic low-grade inflammatory response that exacerbates myocardial cell apoptosis and reduces myocardial compliance, thereby creating a vicious cycle of “myocardial injury—neuroendocrine activation—inflammatory infiltration—worsening cardiac function” (25, 26). As the disease progresses, patients experience a continuous decline in cardiac systolic and diastolic function, a marked reduction in exercise tolerance and quality of life, and persistently high rates of recurrent hospitalisation and long-term mortality. At present, there remains a lack of curative treatments in clinical practice capable of completely halting the progression of heart failure. How to effectively suppress neuroendocrine dysregulation, mitigate myocardial inflammatory damage, reverse ventricular remodelling, and improve long-term patient prognosis has consistently been a key focus and challenge in clinical research on heart failure (27).

Current standard clinical management of heart failure primarily consists of basic symptomatic treatment, centring on classic medications. These can, to a certain extent, alleviate patients' clinical symptoms, reduce the cardiac workload and delay the decline in cardiac function, and thus form the cornerstone of heart failure treatment (28). However, long-term clinical practice has revealed that some patients continue to experience persistent deterioration in cardiac function, recurrent episodes of the disease, and a relatively high incidence of adverse events following standard treatment, with overall long-term prognostic outcomes remaining suboptimal (29). Therefore, the exploration of novel therapeutic regimens that combine myocardial protection, anti-inflammatory effects, inhibition of neuroendocrine activation, and reversal of ventricular remodelling is of significant importance for optimising the heart failure treatment system and enhancing clinical efficacy and drug safety (30). Accordingly, this study employed sacubitril-valsartan and SGLT2 inhibitors to treat heart failure patients, with the aim of clarifying the clinical value of these agents—either in combination or when used alone—in improving cardiac function, serological markers, quality of life and safety. The study aims to provide evidence-based support to optimisation to clinical management of heart failure.

Baseline findings from this study indicate that there were no noticeable discrepancies observed between both groups pre-treatment (*P* > 0.05). These results confirm that the baseline data enrolled in two groups were balanced and comparable. This reduces the risk of confounding related to measured baseline variables. However, residual confounding from unmeasured factors cannot be completely excluded in this retrospective observational study. Despite this limitation, the balanced baseline still provides a reliable preliminary foundation for subsequent objective comparisons of the intervention effects of the two treatment regimens on cardiac function, serological indicators, life quality and safety in heart failure patients, thereby ensuring the authenticity and scientific validity of the intergroup comparison results in this study.

In terms of key indicators of cardiac structure and function, LVEF, LVEDD, LVESD and CO levels were essentially consistent between both groups pre-treatment, with no marked discrepancy observed between groups (*P* > 0.05). Following clinical intervention, both groups indicated varying degrees of improvement in cardiac function and ventricular structural parameters. Furthermore, the SS group exhibited remarkably lower LVEDD and LVESD values to SV group, whilst LVEF and CO levels were remarkably higher in SS group to SV group (*P* < 0.05). The decrease in LVEDD and LVESD indicates a reduction in end-diastolic and end-systolic volume of the left ventricle, directly reflecting that ventricular dilatation has been effectively curbed and the process of ventricular remodelling has been remarkably inhibited (31, 32). As core indicators for evaluating myocardial contractile function and cardiac pumping efficiency, elevated levels of LVEF and CO indicate enhanced myocardial contractility and improved overall cardiac pumping function (33). The above results fully demonstrate that, compared with the SV treatment regimen, the SS regimen possesses more pronounced clinical advantages in reversing pathological ventricular remodelling, repairing cardiac structure, and enhancing myocardial contractile function and circulatory perfusion capacity in heart failure patients. Gu et al. reported improvements in cardiac function in a study of elderly heart failure patients and reduced ejection fraction treated with sacubitril-valsartan; both LVEDD and LVESD decreased remarkably following treatment, which is similar to the findings to this study (34).

A 5-year retrospective cohort study conducted by Koçak et al. further confirmed that monotherapy with sacubitril and valsartan can lead to sustained increase in LVEF, decrease in NT proBNP, improvement in NYHA functional grading, and reduction in readmission rates for heart failure (35). The improvement in LVEF in this study is similar to the early change level reported in this report. On this basis, this study further confirms that the addition of SGLT2 inhibitors to sacubitril-valsartan can bring additional benefits to cardiac structure, neuroendocrine, and inflammatory indicators beyond monotherapy, providing supplementary real-world data for existing longitudinal observational evidence.

With regard to changes in neuroendocrine activation levels, there were no significant intergroup discrepancies in heart failure-specific markers levels such as serum β-EP, CT-1 and NT-proBNP in either group pre-treatment (*P* > 0.05). The levels of neuroendocrine mediators in both groups decreased remarkably compared with pre-treatment levels, and the reduction in β-EP, CT-1 and NT-proBNP in SS group was remarkably greater than SV group post-treatment (*P* < 0.05). NT-proBNP is a key marker reflecting increased ventricular pressure load and the extent of myocardial damage; a decrease in its levels directly indicates a reduction in cardiac volume and pressure loads (36). CT-1 can continuously mediate the processes of myocardial fibrosis and ventricular remodelling (37), whilst excessive expression of β-EP exacerbates myocardial damage and inhibits myocardial contractile function (38). These findings suggest that combination therapy may suppress excessive neuroendocrine activation in heart failure patients, which could contribute to reduced neuroendocrine-mediated myocardial injury and slower disease progression. This may represent one potential mechanism underlying the observed cardiac functional benefits, although the exact molecular pathways cannot be confirmed by circulating biomarker measurements alone.

With regard to the regulation of the body's inflammatory response, there were no noticeable discrepancies in the baseline levels of classic inflammatory factors of both groups pre-treatment (*P* > 0.05). Following treatment, the inflammatory factors levels were remarkably reduced in both groups, and SS group demonstrated a remarkably greater reduction in all inflammatory markers compared to SV group (*P* < 0.05). Chronic low-grade inflammation is a critical pathological pathway in the progression of heart failure. The inflammatory markers can exacerbate ventricular remodelling and worsen cardiac function by inducing inflammatory infiltration of the myocardium, promoting myocardial cell apoptosis and accelerating myocardial fibrosis (39, 40). Our results indicate that combination therapy is associated with greater reductions in circulating inflammatory markers, which may create a more favourable milieu for cardiac structural and functional recovery. Whether this reflects a direct anti-inflammatory effect of the drugs or is secondary to improved haemodynamics cannot be determined from the present data. In their study on cardiac functions in elderly patients with acute myocardial infarction treated with a combination of dapagliflozin and sacubitril-valsartan, Meng et al. reported that inflammatory factor levels also decreased remarkably following combination therapy, a finding consistent with the conclusions of this study (41). A recent meta-analysis pooling nine key RCTs demonstrated the pleiotropic cardioprotective effects of SGLT2 inhibitors in heart-failure patients, regardless of diabetes status. These agents suppress RAAS activation, mitigate myocardial fibrosis, and optimize myocardial energy metabolism to attenuate pathological ventricular remodelling. Its pooled results also confirmed that SGLT2 inhibitors significantly reduce risks of heart-failure-related hospitalization, cardiovascular death and all-cause mortality (42).

Analysing the results from the perspective of overall clinical efficacy and improvements in patients' quality of life, this study shows that the overall clinical response rate in SS group was remarkably above to SV group (*P* < 0.05). Furthermore, whilst there was no marked discrepancy observed in MLHFQ quality of life scores of both groups pre-treatment, both groups experienced improvements in life quality post-treatment, with MLHFQ scores in SS group being remarkably below to SV group (*P* < 0.05). The increase in the clinical response rate clearly demonstrates that the SS group's treatment regimen is more effective in alleviating clinical symptoms in heart failure patients and enhancing overall treatment outcomes. The reduction in MLHFQ scores indicates a significant alleviation of heart failure-related symptoms, with a marked reduction in the negative impact of the disease on patients' daily lives, psychological well-being and social activities. It is evident, therefore, that the SS group's treatment regimen can effectively improve patients' subjective quality of life whilst simultaneously enhancing objective cardiac function indicators, thereby achieving dual benefits in terms of objective efficacy and subjective prognosis.

Notably, our safety analyses showed numerically lower complication and adverse-reaction rates in the SS group compared with the SV group. Nevertheless, these safety-related observations should be interpreted with caution, given the relatively small absolute number of complication events, the retrospective study design, and the moderate sample size of our cohort. As heart failure patients require long-term regular medication, treatment safety remains critical for ensuring medication adherence and sustaining therapeutic benefits (43). Although our data point towards a favourable safety profile for combination therapy, we cannot draw definitive conclusions that combination treatment reduces cardiovascular complication risk. Larger-scale real-world cohorts are needed to further validate these safety findings before firm inferences for long-term clinical application can be made.

This study presents certain limitations. First, this single-centre retrospective controlled study recruited all participants from one healthcare institution, which limits population diversity. The external validity and generalisability of the findings therefore require further validation in multicentre, large-scale studies. Second, the observation period was relatively short. Serum biomarkers were measured at 3-months after initiation of treatment, and echocardiographic cardiac-function parameters were assessed at the 6-month follow-up after initiation of treatment. Long-term cardiovascular endpoints, rehospitalisation rates and sustained ventricular remodelling beyond 6 months were not evaluated, so the durability of treatment effects requires further investigation. Third, stratified subgroup analyses were not performed. Subgroup stratification by drug dosage, heart failure subtype, NYHA class and age was not conducted, and the dose-response relationship among dosage, efficacy and safety could not be fully characterised. In addition, statistical results were presented alongside corresponding outcome indicators rather than in an independent subsection. Given the moderate sample size, multivariate adjustment for potential confounders was not performed; although baseline characteristics including background medications were well balanced, residual confounding from unmeasured factors cannot be excluded. Moreover, only outcome assessors were blinded to group allocation in this retrospective design. Treating clinicians and patients could not be blinded, so residual assessment bias cannot be completely ruled out. In addition, the number of observed cardiovascular complication events was relatively small; caution should be exercised when interpreting between-group differences in complication risk. Future studies may adopt multicentre, large-scale, long-term follow-up designs with extended observation periods focusing on long-term prognostic indicators. The inclusion of subgroup analyses based on different dosages would further clarify the optimal treatment strategy for the SS group, thereby providing more comprehensive and reliable evidence-based medical grounds for personalised and precision therapies of heart failure patients.

## 5 Conclusions

This study provides scientific guidance for optimising treatment regimens for heart failure patients by comparing the clinical outcomes of SV group and SS group. The findings revealed that SS group had marked advantages in improving cardiac structure and function (reducing ventricular diameter, increasing ejection fraction and cardiac output), inhibiting neuroendocrine activation (downregulating β-EP, CT-1, NT-proBNP levels), reducing the body's inflammatory response (reducing IL-1β, IL-6, TNF-α expression), enhancing clinical total response rate, and improving patients’ quality of life. At the same time, they performed better in reducing the risk of complications and adverse reactions. However, he observation period was relatively short, the sample was drawn from a single center, a lack of exploration into the underlying mechanisms of action of the two regimens, and the failure to identify the optimal treatment regimen. Future research should involve multicentre, large-scale clinical trials with long-term follow-up to further validated the efficacy of interventions in different heart failure populations. Furthermore, molecular biology techniques should be employed to elucidate the mechanisms of action, thereby fully validating the clinical value of these approaches and optimising personalised treatment plans for heart failure.

## Data Availability

The original contributions presented in the study are included in the article/Supplementary Material, further inquiries can be directed to the corresponding authors.
